# Acute Kidney Injury in Intensive Care Unit Patients with Coronavirus Disease 2019

**DOI:** 10.5152/TJAR.2022.21306

**Published:** 2022-04-01

**Authors:** Esra Aktiz Bıçak, Mustafa Bıçak, Fikret Salık, Cem Kıvılcım Kaçar, Osman Uzundere, Sedat Kaya, Hakan Akelma

**Affiliations:** 1 Department of Anaesthesiology and Reanimation, Gazi Yaşargil Training and Research Hospital, Diyarbakır, Turkey

**Keywords:** Acute kidney injury, COVID-19, intensive care unit, mortality, pandemic

## Abstract

**Objective:**

The incidence of acute kidney injury during the hospital stay in patients with coronavirus disease 2019 varies between 8% and 17% in studies. This rate is at the highest levels among the critical patient group monitored in the intensive care unit (23% [14-35%]). In this study, we aimed to assess the incidence of acute kidney injury development, effective factors, and clinical outcomes of patients monitored in the intensive care unit due to coronavirus disease 2019.

**Methods:**

A total of 801 patients were analyzed. Patients were divided into 2 groups as those developing acute kidney injury (n = 408) and those not developing acute kidney injury (n = 393). Patients developing acute kidney injury were staged according to the Kidney Disease Improving Global Outcomes criteria.

**Results:**

In all patients, the mortality rate was 65.2%. The mortality rate for those developing acute kidney injury was identified to be high by a statistically significant degree compared to those not developing acute kidney injury. The mortality rate in Kidney Disease Improving Global Outcomes criteria stage 1 was 81.3%, in stage 2 was 88.3%, and in stage 3 was 91.5%. The frequency of diabetes mellitus type 2, coronary artery disease, and chronic obstructive pulmonary disease in the group developing acute kidney injury was found to be statistically significantly higher. We have found positive correlations between acute kidney injury development and age, sex, history of diabetes mellitus, and ferritin levels in the multivariate analysis.

**Conclusions:**

The development of acute kidney injury in intensive care unit patients with coronavirus disease 2019 is associated with increased mortality. Therefore, predisposing factors should be determined and effective treatment strategies should be established in the early period.

## Main Points

Multisystemic pathologies may be observed secondary to the cytotoxic effect and hyper inflammation observed after viremia in coronavirus disease 2019 patients.

The incidence of developing acute kidney injury rate was highest in critically ill patients and this was associated with increased mortality.

Predisposing factors for acute kidney injury development should be researched well, risky patient groups should be closely monitored, and intensive care treatment strategies should be developed to prevent acute kidney injury development.

## Introduction

The acute respiratory syndrome caused by the severe acute respiratory syndrome coronavirus-2 virus, the third coronavirus infection in the last 20 years in humans, was first isolated in the world in December 2019 in Wuhan, China.^1^ The name of the disease caused by the virus was officially given as “Coronavirus disease 2019” (COVID-19) by the World Health Organization on February 11, 2020. 

Symptoms of the disease are frequently observed 2-14 days after exposure to the virus. The most frequent symptoms on admission to the hospital are fever (43.8% on presentation and 88.7% after hospitalization) and cough (67.8%).^2^ Apart from these, symptoms like myalgia and fatigue (11%), headache (8%), rhinorrhea (4%) diarrhea (2%), and chest pain (2%) may be observed.^3^ Symptoms such as rhinorrhea and upper respiratory tract infection-like symptoms in adults are contrarily rarely observed in children. 

The clinics of patients with severe progression of COVID-19 form the background for the development of diffuse alveolar injury and acute respiratory distress syndrome. Additionally, multisystemic pathologies may be observed secondary to the cytotoxic effect and hyper inflammation observed after viremia in patients.

Studies related to COVID-19 display differences according to the patient population. In a review, it is reported that the incidence of developing acute kidney injury (AKI) was 11% (8-17%) during their stay in hospitalized patients with COVID-19, and this rate was highest in critically ill patients (23% [14-35%]).^4^ The multisystem manifestations of COVID-19 occur as a result of a combination of the direct effects of the viral infection and the indirect effects of the body’s significant inflammatory response to the virus.^5^ In addition to the effects of the virus on the respiratory system, it has the ability to use the angiotensin-converting enzyme II (ACE II) to enter endothelial cells.^6^ Proinflammatory angiotensin increases in the environment due to viral inhibition of the ACE II enzyme. This may be a contributing factor to lung injury and multi-organ dysfunction.^7^ Acute kidney injury can occur through several different mechanisms, including acute tubular necrosis induced by sepsis, fluid restriction, rhabdomyolysis, or hypoxia. Furthermore, intrinsic tissue injury by the direct viral invasion of the renal tubular cells, interstitium, or glomeruli has also been proposed. Varying degrees of acute tubular necrosis, lymphocytic infiltration, and viral RNA have been found on postmortem examination of COVID-19 patients, suggesting the direct invasion of kidney tubules.^8^

Studies in recent periods have shown that AKI development is an independent risk factor for mortality in COVID-19.^9,10^ For this reason, we believe it is necessary to assess in detail the predisposing factors and clinical outcomes of AKI development.

In this study, we aimed to retrospectively assess the incidence of AKI development, effective factors, and clinical outcomes of patients monitored in the intensive care unit due to COVID-19 in our hospital acting as a pandemic center. 

## Methods

The study was performed retrospectively in a single center. After receiving permission from the Ministry of Health (15/11/2020) and the hospital ethics committee (12/02/2021-670), the study included 940 patients with positive polymerase chain reaction test from nasopharyngeal and oropharyngeal swabs monitored in our intensive care unit. A total of 138 patients were excluded from the study. A total of 801 patients were analyzed and divided into 2 groups as those developing AKI (n = 408) and those not developing AKI (n = 393). Exclusion criteria for the study included <18 years of age, treatment not concluded at the time of the study, history of chronic renal failure or chronic renal disease, history of renal transplantation, the patients with a diagnosis of diabetic nephropathy, deficiency in accessing data, and hospitalization time <48 hours.

Study data were obtained from patient follow-up forms and electronic medical records by an experienced anaesthesia expert. Demographic data for patients and blood group antigens were recorded. During admission to the intensive care unit, comorbidities, Acute Physiology and Chronic Health Evaluation II score, biochemical parameters, neutrophil/lymphocyte ratios, and platelet values were recorded. The duration of stay in the intensive care unit, duration of hospitalization, and mortality were recorded.

For assessment of renal functions, the creatinine value at the time of admission to the hospital was used as the basal value. Patients developing AKI were staged according to the Kidney Disease Improving Global Outcomes (KDIGO) criteria.^11^

### Statistical Analysis

Statistical analysis used the Statistical Package for the Social Sciences software 16.0 for Windows program (SPSS Inc.; Chicago, IL, USA). Numerical data are expressed as mean and standard deviation, while categorical data are given as frequency and percentage. The Kolmogorov–Smirnov test was used to assess whether non-categorical data abided by normal distribution or not. Comparison of data abiding by normal distribution used the Student’s *t*-test. Comparison of data not abiding by normal distribution used the Mann–Whitney *U* test, with results given as mean ± standard deviation (SD). Comparison of categorical data in the groups used the chi-square test with results given as n%. With the aim of analyzing independent risk factors related to AKI, a multivariate logistic regression model was created presenting the odds ratio and 95% CI. All comparisons accepted *P* < .05 as significant.

## Results

During the study duration, 940 patients were monitored in the intensive care unit. Twenty-eight patients with chronic renal disease, 38 patients with missing data, and 73 patients without finished treatment were excluded from the study. The study was completed with 801 patients (flow diagram).

The demographic and clinical features of patients are shown in Table 1. When the groups were compared in terms of ages, the ages of patients developing AKI were found to be statistically significantly higher (*P* < .001).

The number of patients over 65 years in the group with AKI was found to be higher by a statistically significant degree than in the group not developing AKI (*P* < .001). 

When patients were assessed in terms of sex, the male gender rate was statistically significantly high in the group developing AKI (*P* = .022).

Diabetes mellitus (DM) type 2, heart failure, chronic obstructive pulmonary disease (COPD), coronary artery disease, and hypertension were evaluated as a comorbidity in all patients. When the groups were compared in terms of all comorbidities, there was no difference between the groups. However, when the groups were compared in terms of the frequency of DM type 2, coronary artery disease, and COPD one by one, these diseases were statistically higher in the group developing AKI than the group not developing AKI (*P* = .004, .006, and .045, respectively).

When the patients were assessed in terms of duration in the intensive care unit, there was no statistically significant difference between the groups (*P* = .105).

When patients were assessed in terms of blood group antigens, the most frequently observed blood group in both groups was A Rh (+). There was no statistically significant correlation between AKI development and blood group antigens.

For all patients included in the study, the mortality rate was 65.2% (n = 523). The mortality rate for those developing AKI was identified to be high by a statistically significant degree compared to those not developing AKI (*P* < .001). 

**Figure 1.**
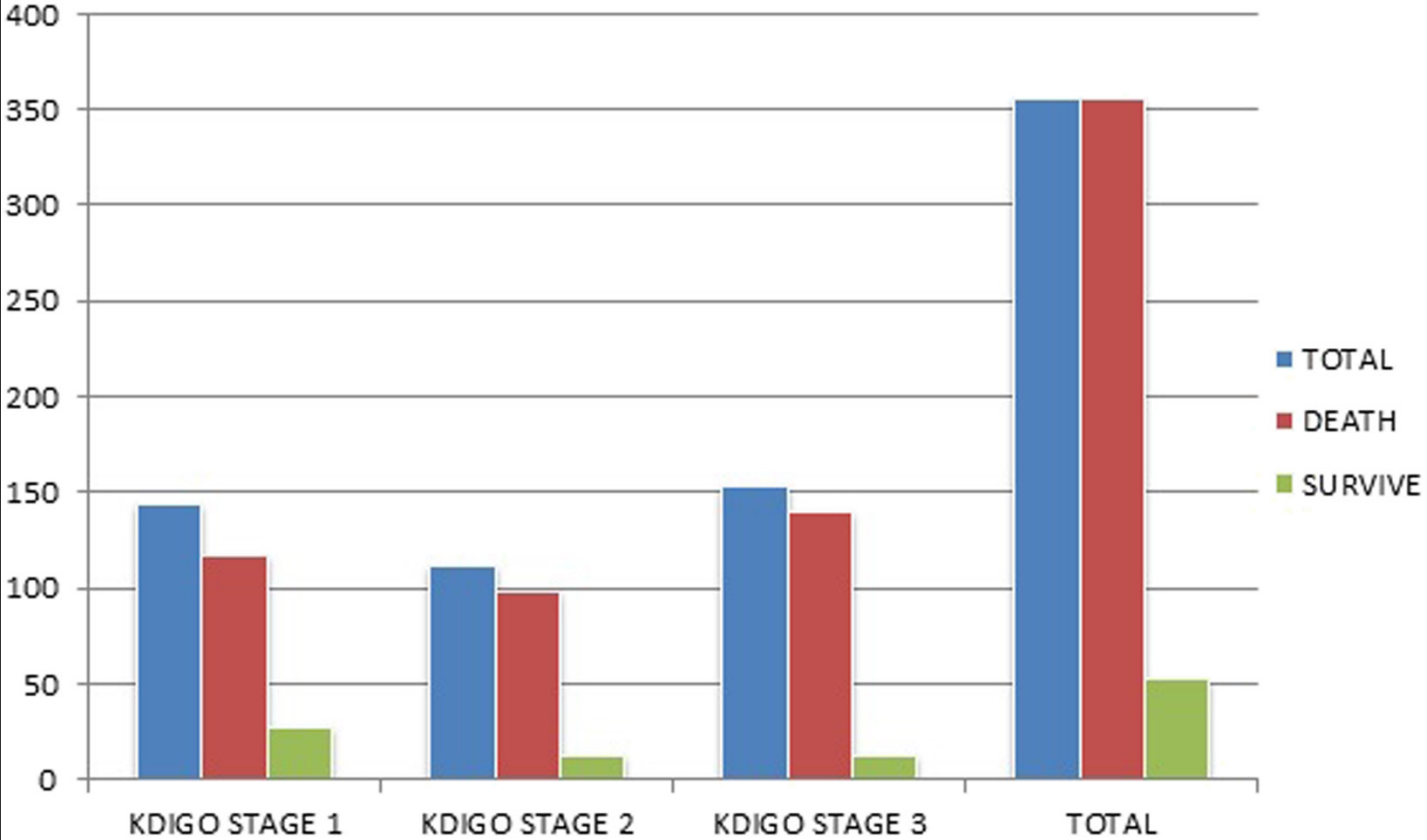
Kidney disease improving global outcomes stages and mortality.

When those developing AKI were assessed in terms of KDIGO stages, 35.3% (n = 144) were stage 1, 27.2% (n = 111) were stage 2, and 37.5% (n = 153) were stage 3. When mortality rates were assessed according to KDIGO staging, the mortality rate in stage 1 was 81.3% (n = 117), in stage 2 was 88.3% (n = 98), and in stage 3 was 91.5% (n = 140) (Figure 1, Table 2). The percentage of patients requiring renal replacement therapy (RRT) was found to be 7.86% (n = 63). Conventional dialysis was applied to 73.0% (n = 46) of the patients who needed RRT, and continuous veno-venous hemodiafiltration was applied to 26.9% (n = 17).

Assessments of patients in terms of complaints on admission are shown in Table 3. The most common complaint on admission to the intensive care unit was identified to be dyspnea. The general status disorder and cough incidence were statistically significantly higher in the group developing AKI (*P* = .004 and .001, respectively).

Laboratory parameters for patients are shown in Table 4. The AKI group had statistically significantly higher white blood cell (WBC), neutrophil, neutrophil/lymphocyte ratio, urea, creatine, lactate dehydrogenase (LDH), potassium, chlorine, lactate, ferritin, d-dimer, and C-reactive protein (CRP) values and lower platelet values.

Multivariate logistic regression analysis was performed to assess factors related to AKI (Table 5). Positive correlations were identified between AKI development with age (aOR [95% CI]: 0.967 [0.964-0.989]), sex (aOR [95% CI]: 0.734 [0.508-0.987]), DM history (aOR [95% CI]: 0.512 [0.381-0.798]), and serum ferritin levels (aOR [95% CI]: 1.000 [0.999-1.000]).

## Discussion

The results of our retrospective study show that there is high acute kidney failure prevalence among patients admitted to the intensive care unit with COVID-19. Acute kidney injury development is associated with higher mortality rates in patients in the intensive care units. According to KDIGO staging, as the stage increases, the mortality rates for patients in intensive care increase.

Studies support that there is higher mortality in advanced age groups and male sex among COVID-19 patients.^9,12,13^ The high mortality in the advanced age group is stated to be associated with anatomic and functional changes observed in the lungs with aging and muscle atrophy resulting in reduced pulmonary reserve, airway clearance, and barrier functions.^14^ In our study, consistent with the literature, we identified higher mortality in the male patient group and patients ≥65 years.

Robbins-Juarez et al^15^ investigated 20 cohort studies including 13 137 patients related to renal disease and found that the AKI prevalence was between 17% and 77%. Rubin et al^16^ found that the AKI development incidence was 80% during the duration patients were admitted to the intensive care unit and identified that the majority of patients developing AKI were KDIGO stage 1. In our study, the AKI prevalence was 49.1%, consistent with the literature. However, contrary to many studies in patients in the intensive care unit, the largest proportion of our patient group was KDIGO stage 3. We associate this with the high prevalence of advanced age, male gender, and high comorbidity in our patient group, which are known to be poor prognostic factors in the development of AKI, and the fact that the majority of our patients were transferred to our clinic from the periphery at a late stage after primary treatment options were applied.^12,14,17^

The proportion of patients needing RRT increased to between 5.2% and 25% at severe COVID-19 infection.^2,17^ When we examined our study results, we found the percentage of patients needing RRT to be 7.86%, in line with the literature.

While 80% of COVID-19 patients had mild symptoms, 2% of patients had severe progression which was observed to cause mortality.^12^ A study of patients in the intensive care unit by Xiaobo Yang et al^17^ stated the most frequent complaints on admission were fever (98%), cough (77%), and dyspnea (65.5%). A study of patients in the intensive care unit by Eastin et al^18^ emphasized that the most common complaints on admission were shortness of breath (76.2%), fever (52.4%), and cough (47.6%).^18^ In our study, the most common complaints were dyspnea (86.9%) and cough (13.4%). Contrary to the literature, the incidence of fever was 6.3%. This difference may be linked to our hospitals being the most comprehensive COVID-19 center in the region and with the frequent transfer of patients monitored and treated in external intensive care units who have already received broad-spectrum antibiotherapy, antiviral, and anti-inflammatory treatment. A statistical difference was observed for general status disorder and cough in the patient group developing AKI compared to those who did not develop AKI. Though involvement potential is primarily in the respiratory system for viral agents like coronavirus, studies show that the central nervous system and many other systems may be involved.^19,20^ We believe more frequent observation of general status disorder in those developing AKI may be associated with more severe progression of the clinic in patients and multisystemic involvement like thrombotic events and the nervous system.^21^

The presence of comorbidities increases the risk of infection with COVID-19 and negatively affects the progression of the disease, causing deaths.^22^ Previous studies stated that diabetic patients had increased morbidity, mortality, and additional poor progression of the disease.^23^ Chronic obstructive pulmonary disease or any other respiratory system disease are emphasized as other poor prognostic factors.^24^ A meta-analysis by Paudel et al^25^ investigating 1786 patients stated that the most common comorbidities in COVID-19 were hypertension (15.8%), cardiovascular diseases (11.7%), and DM type 2 (9.4%).^25^ The COVID-19-Associated Hospitalization Surveillance Network report about 1478 patients hospitalized due to COVID-19 stated that the most common comorbidities were hypertension (49.7%), obesity (48.3%), and chronic lung disease (34.6%).^26^ In our patients, comorbidity incidence was 73.5%. The most commonly observed comorbidities were hypertension (39.1%), DM type 2 (27.0%), and coronary artery disease (16.8%). Patients developing AKI had a statistically significantly higher incidence of DM, coronary artery disease, and chronic lung disease. Our multivariate logistic regression analysis results found the presence of DM was a predisposing factor for the development of AKI. This result is compatible with the literature. In diabetic patients, COVID-19 has more severe progression. Studies have held increased ACE-2, increased furin expression, impaired T-cell function, and increased interleukin-6 responsible for the pathophysiology of severe infection in diabetic patients.^2,27-29^ For this reason, we believe it is necessary to monitor diabetic patients in the intensive care unit due to COVID-19 more carefully for severe infection and renal functions. 

Studies of COVID-19 patients have found a strong association between lymphopenia and disease severity. Additionally, some studies support the view that lymphopenia regulation is an important parameter showing amelioration.^30-33^ Meta-analyses have stated there are associations between low lymphocyte, hemoglobin, and platelets, and increased serum glucose, sodium levels, LDH, WBC, CRP, urea, creatinine, fibrinogen, ferritin, and d-dimer levels with severity of disease in COVID-19 patients.^32,34^ Our study results show that those developing AKI had statistically significantly higher WBC, neutrophil, urea, creatinine, sodium, potassium, lactate, d-dimer, and ferritin levels, and lower lymphocyte and platelet levels. As renal function injury is seen as a component of multisystemic involvement related to severe infection, the prognosis is worsened due to increased tissue injury and perfusion disorder in this patient group. From this aspect, our data are consistent with literature data.

In the broadest scope report related to COVID-19, the mortality rate was 2.3%; however, it was stated to be 49% for severe patients. Development of AKI has been found to be associated with increased hospital mortality, and mortality has been reported to rise up to 80% in this patient group.^35-39^ Similar to the literature, we identified increased hospital mortality rates with the development of AKI in patients monitored for COVID-19 in intensive care. Mortality among our intensive care patients was 65.2% and this was 86.2% among those developing AKI. We identified that mortality increased as the KDIGO stage increased in those developing AKI. Our mortality rates are higher compared to many studies in the literature. We think this elevation may be due to the fact that the majority of our patients in the intensive care unit were transferred in the terminal stage from more peripheral hospitals of primary admission. The majorities of our patients were KDIGO stage 3 and had factors like male gender and advanced ages which are known to be poor prognostic factors in relation to infection which we think contributed to the increased mortality rates.

Studies show that if the renal injury is not diagnosed and treated early, especially in severe disease, 10-times worse outcomes are obtained.^20^ To reduce mortality in those admitted to the intensive care unit with COVID-19 and with severe infection findings, we think it is necessary to perform frequent fluid balance surveillance, close hemodynamic monitoring, in case of need, inotrope support, effective respiratory support in the early period to minimize hypoxic cell injury, suitable nutritional support for the clinic and patient characteristics, routine thrombosis prophylaxis, and treatment with the minimal medication appropriate avoiding medications causing organ injury.^40^

The limitations of our study included the lack of information about the patients’ body weights, urine tests of patients, medical treatment, fluid replacement, inotrope support and pressure values, mechanical ventilation rates contributing to the development of AKI, not evaluating AKI recovery rates, and lack of culture results to exclude secondary infections. In addition, the fact that the study was a single-center cohort study and our hospital being a referral center, which maybe because of the increased mortality, are among the limitations of our study. 

## Conclusion

The development of AKI in ICU patients with COVID-19 is associated with increased mortality. Predisposing factors for AKI development should be researched well, risky patient groups should be closely monitored and intensive care treatment strategies should be developed to prevent AKI development. We believe there is a need for more comprehensive multicenter studies about the topic to support the literature.

### Ethics Committee Approval:

Ethical committee approval was received from the Diyarbakır Gazi Yaşargil Training and Research Hospital (12.02.2021-670).

### Informed Consent:

N/A.

### Peer-review:

Externally peer-reviewed.

### Author Contributions:

Concept and Design – A.B.E., U.O.; Data Collection – B.M., K.C.K., S.F., A.B.E., U.O.; Supervision – K.C.K., A.H., U.O., K.S.; Writing the Manuscript – B.M., AB.E., S.F.; Critical Review – A.B.E., B.M., A.H.

### Declaration of Interest:

The authors have no conflicts of interest to declare.

### Funding:

The authors declared that this study has received no financial support.

## Figures and Tables

**Table 1.** Demographic and Clinical Characteristics of Patients (Mean ± SD)

	Non-AKI (n = 393)	AKI (n = 408)	P
Age (year)	66.3 ± 15.4	71.9 ± 12.4	<.001^*^
>65 age	229 (58.2%)	305 (74.7%)	<.001^*^
Gender (male/female)	198/195	167/241	.022^*^
Comorbidity	289 (73.5%)	301 (73.7%)	.671
Diabetes mellitus type 2	90 (22.9%)	127 (31.5%)	.004^*^
Heart failure	14 (3.5%)	19 (4.7%)	.821
Chronic obstructive pulmonary disease	31 (7.8%)	41 (10.1%)	.045^*^
Hypertension	153 (38.9%)	161 (39.9%)	.930
Coronary artery disease	56 (14.2%)	79 (19.6%)	.006^*^
Intensive care ünit days	11.6 ± 8.7	10.9 ± 9.2	.105
Mortality in ICU	168 (42.7%)	355(87.0%)	<.001^*^
*Statistically significant. AKI, acute kidney injury; ICU, intensive care unit.			

**Table 2.** Mortality of Patients in Subgroups

	Total (n = 801)	Mortality (n = 523)
Non-AKI	393	168 (42.7%)
AKI	408	355 (87.0%)
KDIGO 1	144	117 (81.2%)
KDIGO 2	111	98 (88.2%)
KDIGO 3	153	140 (91.5%)
RRT need	63	57 (90.5%)
CD	46	42 (91.3%)
CVVHDF	17	15 (88.2%)

AKI, acute kidney injury; KDIGO, Kidney Disease Improving Global Outcomes; RRT, renal replacement therapy; CD, conventional hemodialysis; CVVHDF, continuous venovenous hemodiafiltration.

**Table 3.** Symptoms at the Time of Application

	Non-AKI (n = 393)	AKI (n = 408)	*P*
Fever	26 (6.6%)	25 (6.1%)	.571
Cough	64 (16.2%)	44 (10.7%)	.001^*^
General status disorder	49 (12.4%)	56 (13.7%)	.004^*^
Dyspnea	341 (86.7%)	356 (87.2%)	.833
Other	23 (5.8%)	8 (1.9%)	.850

*Statistically significant. AKI, acute kidney injury.

**Table 4.** The Comparison of Patients in Terms of Laboratory Values at the Time of First Admission to ICU (Mean ± SD)

	Non-AKI (n = 393)	AKI (n = 408)	*P*
WBC, ×10^9^ L^-1^	11.7 ± 6.9	12.9 ± 7.6	.010^*^
Neutrophil, ×10^9^ L^-1^	9.7 ± 5.2	11.1 ± 6.6	.003^*^
Lymphocytes, ×10^9^ L^-1^	1.3 ± 4.5	1.2 ± 2.9	.184
Neutrophil/lymphocytes	12.8 ± 12.2	15.2 ± 14.3	.004^*^
Platelets, ×10^9^ L^-1^	261.5 ± 107.3	245.4 ± 100.0	.036^*^
Hemoglobin, g L^-1^	12.9 ± 1.9	12.8 ± 2.2	.174
Urea, mg dL^-1^	48.6 ± 31.9	77.9 ± 51.0	<.001^*^
sCr, mg dL^-1^	1.05 ± 1.18	1.69 ± 1.43	<.001^*^
ALT, U L^-1^	51.6 ± 146.4	60.7 ± 232.8	.136
AST, U L^-1^	72.3 ± 225.8	96.3 ± 349.8	.219
LDH, U L^-1^	506.9 ± 379.1	594.2 ± 793.3	.021^*^
Na, mEq L^-1^	136.6 ± 6.0	137.8 ± 7.9	.225
K, mEq L^-1^	4.2 ± 0.6	4.3 ± 0.7	.001^*^
Cl, mEq L^-1^	102.4 ± 6.5	104.3 ± 8.0	.001^*^
Lactate, mmol L^-1^	2.8 ± 2.6	3.2 ± 2.6	.002^*^
Ferritin, μg L^-1^	748 ± 686	980 ± 833	<.001^*^
d-dimer, ng mL^-1^	1365 ± 3127	2327 ± 5065	<.001^*^
CRP, mg L^-1^	136.6 ± 86.8	148.7 ± 87.2	.032^*^

*Statistically significant. WBC, white blood cell; sCr, serum creatinine; ALT, alanine aminotransferase; AST, aspartate aminotransferase; LDH, lactate dehydrogenase; Na, sodium; K, potassium; Cl, chloride; CRP, C-reactive protein.

**Table 5.** Multivariate Logistic Regression Analysis of Factors Associated with AKI

Characteristics	95% CI			*P*
OR	Lower	Upper
Age	0.967	0.964	0.989	<.001^*^
Sex (male)	0.734	0.508	0.987	.060
Diabetes mellitus type 2	0.512	0.381	0.798	<.001^*^
Coronary artery disease	0.618	0.421	1.002	.024
WBC	1.011	0.966	1.050	.615
Neutrophil	0.946	0.917	1.020	.037^*^
Lactate	1.000	0.959	1.088	.988
Ferritin	1.000	0.999	1.000	.001^*^
d-dimer	1.000	1.000	1.000	.101
CRP	1.000	0.998	1.002	.817
Na	0.987	0.971	1.020	.242
Hypertension	1.231	0.873	1.750	.229
Chronic obstructive pulmonary disease	1.581	0.871	2.564	.089
PLT	1.002	1.000	1.003	.022^*^

*Statistically significant. WBC, white blood cell; CRP, C-reactive protein; Na, sodium; PLT, platelet.
